# Use of Animal Models in Molecular Imaging

**DOI:** 10.1155/2020/5168147

**Published:** 2020-02-14

**Authors:** Aage Kristian Olsen Alstrup, Svend Borup Jensen, Pia Afzelius, Pedro Neto, Michael Pedersen

**Affiliations:** ^1^Aarhus University Hospital, Aarhus, Denmark; ^2^Aalborg University Hospital, Aalborg, Denmark; ^3^Department of Chemistry and Biosciences Aalborg University, Aalborg, Denmark; ^4^Nordsjællands Hospital, Hillerød, Denmark; ^5^McGill University, Montreal, Canada

Modern preclinical research is continuously using imaging techniques to provide scientific results useful for human medicine. The imaging techniques provide either morphological or volumetric presentations of the organs of interest, hemodynamic measures of the cardiovascular system, and/or semiquantitative/empirical parameters of the cellular metabolism and function [1]. Disease progression is followed noninvasively over time in experimental animal models, providing information about pathophysiologic characteristics that mimic human diseases [2]. In parallel, focus emerges regarding reduction of both suffering and the number of experimental animals used per study, in accordance with the principles behind 3 Rs for good animal ethics in research: replacement, reduction, and refinement [3].

Experimental animals must be situated in a fixed position and respiratory and physiologically stable to ensure imaging with optimal quality [4, 5]. Nonetheless, little attention has been paid to the impact of anesthesia, sex, choice of species and strain/stock, housing conditions, diet/fasting, behavioral scores, circadian rhythm, etc, on the acquired data [6]. Consequently, the rate of successful translation from animal models to human diseases is modest. This failure to translate from animals to humans is likely due, in part, to poor methodology and failure of the models to accurately mimic the human disease condition [7]. However, a deeper insight into the abovementioned parameters could likely improve the translation ability of preclinical animal models. Therefore, we need to address these fundamental issues in experimental animal research.

This special issue in Contrast Media and Molecular Imaging focuses on these issues, and the emphasis is on the relationship between molecular imaging measures and the impact of factors.

In one of the published studies, M. D. Overgaard and coworkers investigated the placental uptake of a radiolabeled tracer. The authors recognized that the biological differences in the placenta between laboratory chinchillas and humans made it difficult to transfer the results. Another research team who also used pregnant chinchillas had more luck. Using ultrasound, A. Greco and coworkers examined the development of the fetuses, including the age of the fetuses and found that pregnant chinchillas are, in this context, useful models for human pregnancy.

The choice of model, as well as experimental animals to mimic humans, was the subject of a PET study comparing dosimetry of a drug performed in mice, pigs, and humans. S. Beykan and coworkers showed that the pig model was a superior model for humans.

In another study, the researchers examined possible pitfalls with imaging of myelin following lysophosphatidylcholine (LPC) injections in the central nervous system in a well-known animal model of demyelination. M. Zhang and coworkers conclusion was that the PET scans advantageously could be supplemented with an MRI scan to avoid the risk of false results due to LPC side effects.

A group of researchers applied an MRI scan for quantifying iron overload in the liver in a mouse model. Y. Matsuo-Tezeka and coworkers found that MRI T2 *∗*  relaxation time was able to determine the content with high sensitivity. In another MRI study perfomed by K. M. Parkins and coworkers and also performed with mice, showed that engineered cells did not form tumors as well as their naïve counterparts, demonstrating the scanner's ability to evaluate animal models.

Furthermore, this special issue includes a review published by G. Musch on molecular imaging techniques to penetrate the depth of the pathophysiological mechanisms at stake during acute pulmonary disease.

*Aage Kristian Olsen Alstrup* *Svend Borup Jensen* *Pia Afzelius* *Pedro Neto* *Michael Pedersen*
